# Oxytosis/Ferroptosis—(Re-) Emerging Roles for Oxidative Stress-Dependent Non-apoptotic Cell Death in Diseases of the Central Nervous System

**DOI:** 10.3389/fnins.2018.00214

**Published:** 2018-04-20

**Authors:** Jan Lewerenz, Gamze Ates, Axel Methner, Marcus Conrad, Pamela Maher

**Affiliations:** ^1^Department of Neurology, Ulm University, Ulm, Germany; ^2^Cellular Neurobiology Laboratory, The Salk Institute for Biological Studies, La Jolla, CA, United States; ^3^Department of Neurology, University Medical Center and Focus Program Translational Neuroscience of the Johannes Gutenberg-University Mainz, Mainz, Germany; ^4^Institute of Developmental Genetics, Helmholtz Zentrum München, Neuherberg, Germany

**Keywords:** programmed cell death, oxytosis, ferroptosis, iron, oxidative stress, brain diseases

## Abstract

Although nerve cell death is the hallmark of many neurological diseases, the processes underlying this death are still poorly defined. However, there is a general consensus that neuronal cell death predominantly proceeds by regulated processes. Almost 30 years ago, a cell death pathway eventually named oxytosis was described in neuronal cells that involved glutathione depletion, reactive oxygen species production, lipoxygenase activation, and calcium influx. More recently, a cell death pathway that involved many of the same steps was described in tumor cells and termed ferroptosis due to a dependence on iron. Since then there has been a great deal of discussion in the literature about whether these are two distinct pathways or cell type- and insult-dependent variations on the same pathway. In this review, we compare and contrast in detail the commonalities and distinctions between the two pathways concluding that the molecular pathways involved in the regulation of ferroptosis and oxytosis are highly similar if not identical. Thus, we suggest that oxytosis and ferroptosis should be regarded as two names for the same cell death pathway. In addition, we describe the potential physiological relevance of oxytosis/ferroptosis in multiple neurological diseases.

## Introduction

Among all of the tissues in the body, the human brain is unique with regard to its complexity. About 86 billion neurons (Herculano-Houzel, 2009) are interconnected to enable an incredible array of responses to exterior stimuli that manifest either as behaviors or as processes that we experience as emotions, thoughts or memories. In addition, the brain is also unique with respect to the longevity of the neuronal cells, which for the most part are as old as the individual (Bhardwaj et al., 2006). With life expectancy increasing worldwide (Robine and Cubaynes, 2017), it is not surprising that diseases characterized by dysfunction of the central nervous system (CNS) like Alzheimer's (AD) and Parkinson's disease (PD) are expected to sharply rise in aging societies. In addition, other neurodegenerative diseases like amyotrophic lateral sclerosis (ALS) or Huntington's disease (HD) pose a tremendous burden both on the patients and the families and caregivers (Domaradzki, 2015; Oh et al., 2015; de Wit et al., 2017). Of note, there are no treatments that stop or reverse any of the aforementioned diseases. Although the pathophysiology of these diseases may vary greatly and the neurons affected show characteristic neuroanatomical distributions, the neurological symptoms caused by the functional impairment are the result of either neuronal dysfunction or death (Herms and Dorostkar, 2016; Handley et al., 2017; Jackson et al., 2017). Neuronal dysfunction, at least experimentally, is reversible (Verity and Mallucci, 2011). However, when neuronal death exceeds a certain threshold, it cannot be functionally compensated for in most areas of the brain (Clement and Belleville, 2010; Malejko et al., 2014). In humans, neurogenesis is thought to occur only in the hippocampus and to a lesser degree in the striatum throughout the lifespan (Bergmann et al., 2015). However, even this has been questioned recently (Sorrells et al., 2018). Consequently, a better understanding of how neurons actually die during neurodegenerative diseases could pave the way for treatments whose ultimate goal is not just to rescue neurons from death but also to re-establish proper neuronal function in these rescued neuronal populations.

In stark contrast to its relevance, there is surprisingly little knowledge about the exact mechanism(s) that underlie neuronal death in different diseases of the brain. Within the last two decades, the dichotomy between apoptosis (previously meaning programmed cell death), and necrosis (referring to an accidental, unregulated cell death) has proven to be an oversimplification as a myriad of regulated cell death subroutines distinct from apoptosis have been described. These include necroptosis (Degterev et al., 2005), parthanatos (Andrabi et al., 2006), oxytosis (Tan et al., 2001a), and ferroptosis (Dixon et al., 2012), to name a few. Yet, the caveat of this new perception is that many of the reports mostly rely on *in vitro* observations. It has proven to be extremely difficult to unequivocally assign which of these different pathways is responsible for neuronal loss in various disease states *in vivo*. The main hurdle to achieving a better understanding of neuronal death processes in different diseases is that neurodegeneration is a very slow process leaving it very challenging to pinpoint neurons that are in the actual process of succumbing to cell death in either autoptic tissues or brains of animal models of CNS diseases. In addition, with the exception of apoptosis (Kerr et al., 1972) and necroptosis (Sun et al., 2012), no established morphological or biochemical markers are available for these different types of cell death. In this review, we describe the historical aspects of the discovery of two highly related, if not identical, forms of regulated cell death, oxytosis and ferroptosis, and summarize the current state of knowledge with respect to their relevance for neuronal death in diseases of the CNS.

## Regulated cell death—apoptosis and beyond

Apoptosis, the first described form of regulated cell death, has long been used as a synonym for all forms of regulated cell death including programmed cell death during development. Morphologically, apoptosis is characterized by membrane blebbing, nuclear condensation and fragmentation and cell shrinkage (Kerr et al., 1972). Initiated either by the intrinsic pathway, which includes mitochondrial cytochrome C release and subsequent activation of caspase 9 (reviewed by Fuchs and Steller, 2011) or by extrinsic stimuli, which involves the activation of death receptors by their cognate ligands, tumor necrosis factor α (TNFα), TRAIL, and FasL, and subsequent activation of caspase 8 (reviewed by Christofferson and Yuan, 2010), apoptosis is executed by the downstream activation of caspases 3 and 7. These, in turn, cleave several hundred different cellular target proteins (reviewed by Luthi and Martin, 2007), resulting in the typical morphological changes and biochemical hallmarks of apoptosis, e.g., 200 bp internucleosomal DNA fragmentation by the caspase-activated DNase (CAD)/DFF40 nuclease (Wyllie, 1980; Liu et al., 1997). In addition, the Kroemer laboratory purified a protein called apoptosis-inducing factor (AIF) that in a caspase-independent manner induces apoptosis-specific structural nuclear changes associated with large scale DNA fragmentation rather than internucleosomal DNA cleavage (Susin et al., 1999).

Almost 30 years ago, Laster and colleagues were the first to observe that TNFα-induced cell death in some sensitive cell lines and under certain conditions is distinct from apoptosis (Laster et al., 1988). This type of cell death was later termed necroptosis (Degterev et al., 2005). It was repeatedly shown that cells undergo this type of regulated cell death when apoptosis is pharmacologically/genetically impeded (Vercammen et al., 1998a,b; Holler et al., 2000; Conrad et al., 2016). Necroptosis requires the kinase activity of receptor-interacting protein 1 (RIP1; also known as RIPK1) and RIP3 (also known as RIPK3), which - among others - are the central components of a multi-protein complex called the necrosome (Vanden Berghe et al., 2016). Downstream of necrosome formation, RIPK3-mediated mixed lineage kinase domain-like protein (MLKL) phosphorylation (Wang et al., 2014), translocation and association of phosphorylated MLKL with phosphatidylinositol phosphates in the plasma membrane, pore formation and influx of calcium ions have been reported to be critical events in the execution of necroptosis (Dondelinger et al., 2014; Wang et al., 2014).

Alternatively, activation of poly(ADP-ribose) polymerase 1 (PARP1) may lead to another distinct, non-apoptotic cell death paradigm called parthanatos (Andrabi et al., 2006; Jouan-Lanhouet et al., 2012). It has been hypothesized that poly-(ADP-ribosyl)-polymers (PARs) generated by abnormal PARP1 activity either modify nuclear proteins or act as signaling molecules to modify proteins in other compartments such as AIF in the mitochondria or hexokinase in the cytosol (Smulson et al., 2000; Andrabi et al., 2014). Experimentally, parthanatos was reported to be triggered in response to the alkylating agent N-methyl-N′-nitro-N-nitrosoguanidine in fibroblasts as well as UV light, ionizing radiation and during excitotoxic cell death in neurons (Yu et al., 2002).

## Oxytosis—regulated cell death induced by glutathione depletion

In the same year when non-apoptotic regulated cell death was observed in response to TNFα (Laster et al., 1988), Murphy and colleagues from Coyle's group reported that in the N18-RE-105 neuroblastoma X retina cell line, glutamate as well as the glutamate analogs quisqualate and ibotenate induced a calcium-dependent form of delayed cell death (Murphy et al., 1988). This type of cell death was associated with depletion of intracellular glutathione (GSH) as it was exacerbated by low cystine medium (a GSH precursor), characterized by increased oxidative stress and inhibited by lipophilic antioxidants (Miyamoto et al., 1989). It was also observed in immature hippocampal neurons (Murphy et al., 1990). The mechanistic link between glutamate exposure and GSH depletion proved to be glutamate-mediated inhibition of cystine import (Murphy et al., 1989). Already at this point, it became apparent that the transport system responsible for glutamate-inhibitable cystine uptake shared similarities with system ${\text{x}}_{\text{c}}^{-}$, a cystine glutamate antiporter originally characterized by Bannai and Kitamura (1980). Although a similar type of cell death was also reported in rat PC12 pheochromocytoma cells (Schubert et al., 1992), most of the subsequent studies addressing this type of glutamate toxicity—initially called oxidative glutamate toxicity—were carried out in HT22 cells, a subclone of the hippocampal cell line HT4 (Morimoto and Koshland, 1991) that was specifically selected for its sensitivity to glutamate (Davis and Maher, 1994). Using this cell-based model, it was possible to characterize in detail the biochemical events that sequentially lead to cell death. It was demonstrated that this type of cell death is associated with a prominent increase in reactive oxygen species (ROS) generation following GSH depletion, which is followed by the final lethal influx of calcium (Tan et al., 2001a). In 2001, oxytosis—a term that highlights both the ROS accumulation that is characteristic of this type of cell death as well as the fact that it is a form of regulated cell death distinct from apoptosis—was coined for this new form of non-apoptotic regulated cell death (Tan et al., 2001a).

## Ferroptosis—an iron-dependent non-apoptotic form of regulated cell death induced by potential anti-cancer drugs

Using a high through-put screen for new genotype-specific anti-cancer therapeutics, Stockwell's group identified in 2003 a novel compound, erastin, which induced non-apoptotic cell death in human transformed foreskin fibroblasts (Dolma et al., 2003). This type of cell death proved to be associated with the loss of mitochondrial integrity and mitochondrial ROS generation in the absence of morphological or biochemical features of apoptosis (Yagoda et al., 2007). Erastin-induced cell death could be blocked by lipophilic antioxidants (Yagoda et al., 2007). Later, the compound RSL3 was identified that induced a similar type of cell death (Yang and Stockwell, 2008). As it could be demonstrated that cells genetically sensitized to this type of cell death show higher levels of intracellular iron (Yang and Stockwell, 2008), and that cell death could be inhibited by structurally different iron chelators, it was named ferroptosis (Yang and Stockwell, 2008; Dixon et al., 2012).

## Are ferroptosis and oxytosis just two names for the same form of regulated non-apoptotic cell death?

The key initiating step in most experimental paradigms for oxytosis is the inhibition of cystine uptake into the cells. Cystine can be imported into cells via four transport systems: excitatory amino acid transporters (EAATs) (Hayes et al., 2005), system b^0,+^, a heterodimer of rBAT with SLC7A9 (Chillaron et al., 2010), a heterodimer of rBAT with AGT1/SLC7A13 that mediates the counter transport of aspartate, glutamate and cystine (Nagamori et al., 2016) and system ${\text{x}}_{\text{c}}^{-}$ (Lewerenz et al., 2013). System ${\text{x}}_{\text{c}}^{-}$ is a heterodimeric amino acid transporter comprising xCT (SLC7A11) and 4F2hc (SLC3A2) as the heavy chain, which specifically transports cystine, glutamate, and the non-proteinogenic amino acid cystathionine (Lewerenz et al., 2013; Kobayashi et al., 2015). The fact that system ${\text{x}}_{\text{c}}^{-}$ inhibition pharmacologically through substrate inhibitors like aminoadipate, homocysteate, and quisqualate (Murphy et al., 1989, 1990; Maher and Davis, 1996) or genetically in cells derived from xCT knock-out mice (Sato et al., 2005) induces cell death indicates that system ${\text{x}}_{\text{c}}^{-}$ inhibition is responsible for the initiation of oxytosis by inhibiting cystine uptake in most cells studied. However, in addition to cystine starvation or inhibition of cystine import, inhibition of GSH synthesis by buthionine sulfoximine (BSO), an inhibitor of glutamate cysteine ligase (GCL), the rate-limiting enzyme in GSH biosynthesis, can induce oxytosis (Li et al., 1998; Ishige et al., 2001b; Lewerenz et al., 2003). This indicates the relevance of GSH depletion for the initiation of oxytosis in cells sensitive to this type of cell death whereas in the presence of high expression of xCT, cystine/cysteine might compensate for the GSH deficiency (Banjac et al., 2008; Mandal et al., 2010).

Most interestingly, the first reported inducer of ferroptosis, erastin (Dixon et al., 2012) is a system ${\text{x}}_{\text{c}}^{-}$ inhibitor (Dixon et al., 2014) and transcriptome changes induced by erastin can be reverted by by-passing cysteine depletion due to system ${\text{x}}_{\text{c}}^{-}$ inhibition by using β-ME in the culture medium (Dixon et al., 2014) similar to xCT KO mice (Sato et al., 2005). Hence, it is reasonable to assume that oxytosis and ferroptosis represent very similar (or even the same) forms of regulated cell death. Therefore, in the following sections we will summarize the similarities and differences and discrepancies for non-apopotic regulated cell death termed either oxytosis or ferroptosis.

## The role of lipoxygenases in the execution of ferroptosis and oxytosis

The series of events leading to cell death by oxytosis following the inhibition of system ${\text{x}}_{\text{c}}^{-}$ or cystine starvation have been quite well-characterized, although some questions and controversies remain. First, GSH levels drop in a time-dependent manner while ROS, as measured by dichlorofluorescein (DCF) fluorescence (a probe that mostly detects hydrophilic ROS; Li and Pratt, 2015), exhibit a linear increase (Tan et al., 1998a). However, when GSH falls below ~20% (6–8 h of glutamate treatment), an exponential increase in ROS levels ensues (Tan et al., 1998a). Subsequent experiments identified 12-lipoxygenase activity (12-LOX) and 12-LOX-mediated peroxidation of arachidonic acid as an important link between GSH depletion and ROS accumulation (Li et al., 1997b). During the induction of oxytosis, the cellular uptake of arachidonic acid is enhanced, 12-LOX activity (measured as the production of ^3^H-12-hydroxyeicosatetraenoic acid (HETE) from ^3^H-arachidonic acid in cell lysates) was increased and LOX proteins were translocated to the plasma membrane. In addition, exogenous arachidonic acid potentiates oxytotic cell death. Currently, the precise LOX responsible for the 12-LOX activity is not clear. HT22 cells do not express ALOX15, ALOX12, or ALOX12b, but only ALOX15B (our unpublished observations and Wenzel et al., 2017). Moreover, murine ALOX15B exhibits almost exclusively 8-LOX activity (Jisaka et al., 1997). Inhibition of LOX activity in HT22 cells by multiple inhibitors with different reported specificities including NDGA, baicalein, CDC, AA-861 and 5,8,11,14-ETYA blocked ROS accumulation and cell death induced by GSH depletion (Li et al., 1997b; Pallast et al., 2009). Interestingly, murine embryonic fibroblasts (MEF) deficient in ALOX15 were protected against BSO-induced cell death (Seiler et al., 2008). Surprisingly, the ALOX5 inhibitor zileuton (Carter et al., 1991) also protected HT22 cells against glutamate-induced oxytosis and ferroptosis induced by erastin (Liu et al., 2015).

A highly similar pharmacological profile was reported for genetically-engineered MEF in which cell death associated with massive lipid peroxidation could be induced via glutathione peroxidase 4 (GPX4) inactivation (Seiler et al., 2008). Here, both linoleic and arachidonic acid exacerbated cell death, while multiple LOX inhibitors (NDGA, AA861, baicalein, PD146176, MK866, MJ33, BWA4C, and zileuton) protected against GPX4 deficiency (Seiler et al., 2008; Friedmann Angeli et al., 2014). A similar array of LOX inhibitors protected against ferroptosis induced by RSL3 in acute lymphatic leukemia cells (Probst et al., 2017). MEFs express ALOX15, ALOX12, ALOX12B, and ALOX5, but not ALOX15B. Whereas ALOX15 deficiency alone did not rescue cell death induced by GPX4 inactivation in MEFs, additional knock-down of ALOX5 did delay cell death (Friedmann Angeli et al., 2014). The human fibrosarcoma cell line HT1080 expresses ALOX15, ALOX12, ALOX12B, ALOX5, ALOX15B, and ALOXE3 (Yang et al., 2016). In these cells, knock-down of both ALOX15B and ALOXE3 was sufficient to recapitulate the protective effect of pharmacological LOX inhibition against erastin-induced cell death (Yang et al., 2016). In G-401 rhabdoid tumor cells, which express all LOX isoforms, knock-down of all of these protected against ferroptosis induced by system ${\text{x}}_{\text{c}}^{-}$ inhibition, but not against RSL3-induced ferroptosis (Yang et al., 2016). Moreover, when deuterated linoleate was incorporated into the membranes of these cells, they became resistant to ferroptosis (Yang et al., 2016). Deuterated polyunsaturated fatty acids have deuterium in place of the bis-allylic hydrogens, which slows the initiation of deuterium abstraction and subsequent radical generation (Shchepinov et al., 2011). In addition, a p53-induced increase in spermidine/spermine N1-acetyltransferase sensitizes cells to tert-butylhydroperoxide-induced ferroptosis via transcriptional induction of ALOX15 (Ou et al., 2016). Using redox phospholipidomics, Kagan et al. showed that double- and triple-oxygenated arachidonic and adrenic acid-containing phosphatidylethanolamine species with C18 fatty acids (C18:0 or C18:1) at the sn-1 position and C20:4 or C22:4 fatty acids at the sn-2 position are the preferential substrates for LOX in ferroptosis induced by RSL3 or GPX4 deficiency in MEF (Kagan et al., 2017). Similar oxygenated lipid species were found to be generated in cell lysates by human recombinant ALOX15. Moreover, it was proposed that vitamin E species as well as the ferroptosis inhibitors liproxstatin-1 and ferrostatin-1 inhibit LOX (Wenzel et al., 2017; Zilka et al., 2017). However, as both liproxstatin-1 and ferrostatin-1 inhibit ferroptosis at concentrations not high enough to inhibit human ALOX15 overexpressed in HEK cells, it was concluded that their ability to protect against ferroptosis is independent of LOX inhibition.

**Figure 1:**
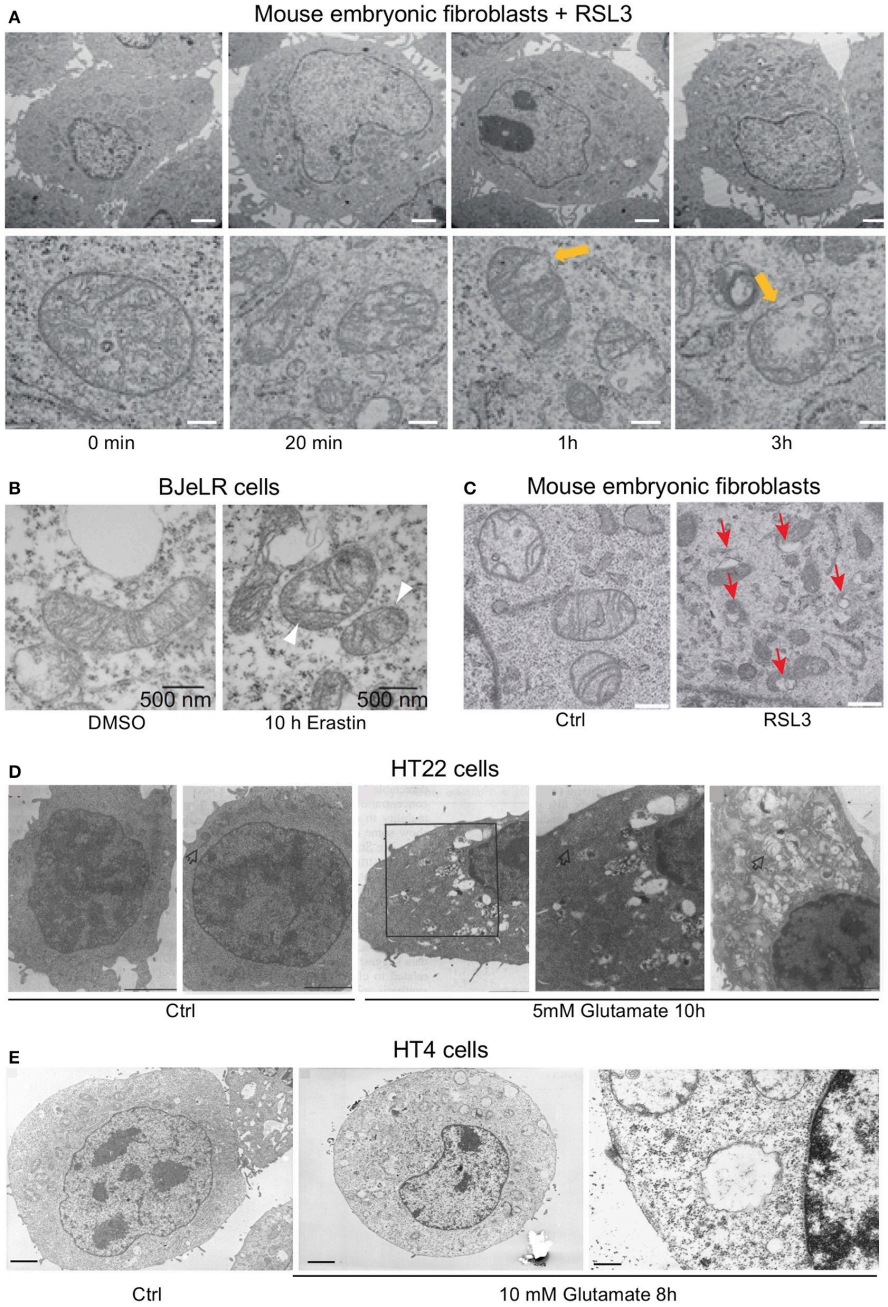
Ultrastructural changes in cells undergoing ferroptosis and oxytosis comprise prominent but diverse mitochondrial abnormalities while nuclear integrity is preserved. Electron micrographs of **(A)** mouse embryonic fibroblasts (MEF) showing a time-dependent outer mitochondrial membrane rupture (yellow arrows) upon ferroptosis induction using RSL3 (50 nM; scale bars 2 μm top row, 200 nm bottom row) while nuclear integrity is preserved, **(B)** of BJeLR cells treated with DMSO (10 h) or erastin (37 μM, 10 h) showing shrinkage and increased electron density of the mitochondria. **(C)** Similar morphological changes as in **(B)** in response to RSL3 in MEF. **(D)** HT22 cells after control treatment (panels 1/2) and after 5 mM glutamate for 10 h (panels 3–5). Panels 3/4: low- and high-power micrographs, respectively, of the same region of the same cell. Arrows indicate mitochondria. Bars in panel 1 = 5 μm; panels 2, 3, and 5 = 2 μm; and panel 4 = 1 μm. Glutamate-induced oxytosis is also characterized by preserved nuclear structure in addition to prominent swelling of the endoplasmic reticulum, Golgi apparatus and mitochondria as well as cytoplasmic vacuolization. Mitochondria showed loss of the cristae. **(E)** Oxytosis induced by glutamate in HT4 cells. (**E**, left) HT4 cells with no glutamate. Mitochondria have a regular shape and optical density. (**E**, middle and right) glutamate-treated cell (10 mM for 8 h). The mitochondria appeared to be swollen and degraded with low optical density. Mitochondrial outer membrane disruption is observed. Scale bars 1.85 μm (left and middle), 0.21 μm (right). [Modified and reproduced with permission from **(A)** (Friedmann Angeli et al., 2014), **(B)** (Dixon et al., 2012), **(C)** (Doll et al., 2017), **(D)** (Tan et al., 1998b), and **(E)** (Tirosh et al., 2000)].

The activity of acetyl-CoA synthetase long-chain member 4 (ACSL4) was shown to be essential for cells undergoing ferroptosis (Doll et al., 2017). ACLS4 knock-out cells accumulate only free oxidized polyunsaturated fatty acids (PUFAs), indicating that esterified oxidized PUFAs contribute to the generation of proximate signals of ferroptosis (Kagan et al., 2017). Recently, it was proposed that when ALOX15 or ALOX15B form complexes with the promiscuous small scaffolding protein phosphatidylethanolamine-binding protein 1 (PEBP1), their ability to oxidize PUFAs shifts from free PUFAs to esterified PUFAs thereby inducing the generation of phosphatidiylethanolamine lipid hydroperoxides (Wenzel et al., 2017). Most interestingly, ALOX15 has been reported to be involved in programmed organelle degradation by binding to intracellular membranes of various organelles while sparing the plasma membrane (van Leyen et al., 1998) and ALOX15 was further shown to bind to mitochondria *in vitro* causing membrane disintegration and ROS generation (Pallast et al., 2009). This is consistent with the disintegration of intracellular organelles observed in oxytosis (Tan et al., 1998b; Tirosh et al., 2000) and ferroptosis (Dixon et al., 2012; Friedmann Angeli et al., 2014; Doll et al., 2017) (Figure 1).

In summary, most of these results indicate that oxytosis and ferroptosis rely on LOX activity for the initiation of maximal ROS production, which ultimately leads to cell death. Although the specific LOX(s) involved likely depend on the cell type, the current data support a model where GSH depletion leads to the activation of LOX, which then interacts with PEBP1 and binds to membranes, especially of intracellular organelles, where the cell death propagating lipid-ROS, oxidized phosphatidylethanolamines containing arachidonic or adrenic acid, are formed.

## Glutathione peroxidase 4—a key player in ferroptosis, is also relevant in oxytosis

The oxidized phosphatidylethanolamines that accumulate following activation of LOXs can be converted to their respective alcohols by GPX4 (Ursini et al., 1982) and thereby detoxified. As GPx4 depends on an adequate supply of GSH for its activity (Cozza et al., 2017), GSH depletion during oxytosis can be expected to impair GPX4 activity. Of note, the ferroptosis inducer erastin and GSH synthesis inhibitor BSO both induce GSH depletion associated with loss of GPX4 activity in cells sensitive to ferroptosis (Yang et al., 2014). As already mentioned above, genetic inactivation of GPX4 leads to the accumulation of lipid-ROS and a cell death pathway in MEF that shows a very similar pharmacological profile to oxytosis in HT22 cells (Seiler et al., 2008). RSL3, originally identified as a compound that induced cell death similar to that induced by erastin (Yang and Stockwell, 2008), was subsequently found to covalently interact with the active site selenocysteine of GPX4 to inhibit its enzymatic activity (Yang et al., 2016).

Overall, it seems that ultimately the inactivation of GPX4 leads to the same consequences as activation of LOX: accumulation of lipid-ROS in the inner leaflet of biomembranes. When system ${\text{x}}_{\text{c}}^{-}$ is inhibited *in vitro*, GSH depletion leads to activation of LOX and inactivation of GPX4, both of which are involved in oxytosis and ferroptosis. In addition, both processes might be linked as LOX activation requires the oxidation of its ferric iron to ferrous iron. This is facilitated when phospholipid hydroperoxides accumulate upon GPX4 inhibition (Conrad et al., 2007).

## ROS generation in oxytosis and ferroptosis

ROS generation is an essential step in oxytosis indicated by the fact that the lipophilic antioxidant α-tocopherol is an efficient oxytosis inhibitor (Ishige et al., 2001b). In the initial phase of glutamate-induced oxytosis in HT22 cells, ROS levels increase, as detected by DCF fluorescence (Tan et al., 1998a). Although LOX might directly produce ROS, the major source of ROS during the exponential phase of accumulation appears to be complex I of the mitochondrial electron transport chain (Tan et al., 1998a). Along with DCF-detectable ROS accumulation, excessive mitochondrial superoxide production, as indicated by increased MitoSOX fluorescence, was also detected (Liu and Schubert, 2009). The mechanism of ROS production was identified as reverse electron transfer at the flavin mononucleotide group of complex I that could be prevented by the flavin protein inhibitor DPI in parallel with the inhibition of ROS production as detected by both ROS probes (Liu and Schubert, 2009). The necessity of mitochondrial ROS production for the execution of oxytosis in HT22 cells is exemplified by the observations that DPI as well as the mitochondrial uncoupler cyanide p-trifluoromethoxyphenylhydrazone (FCCP) protect nerve cells from oxytosis (Tan et al., 1998a; Liu and Schubert, 2009). In addition, high concentrations of the monoamine oxidase inhibitor clorgyline also block ROS production from complex I and cell death (Tan et al., 1998a) (Table 1). NADPH oxidase Nox4 (Ha et al., 2010) and lysosomes (Kubota et al., 2010) may also contribute to oxidative stress in oxytosis. However, although the accumulation of large amounts of intracellular ROS is thus a necessary step in oxytosis, it is not sufficient to cause death as cell death can be inhibited downstream of ROS accumulation (Li et al., 1997a; Ishige et al., 2001b).

**Table 1:** Oxytosis and ferroptosis show an identical pharmacological profile.

**Compound**	**Cell survival (%)**		
	**Oxytosis**	**Ferroptosis**	
	**Glutamate**	**Erastin**	**RSL3**
No treatment	13.5	10.0	12.2
Clorgyline (100 μM)	86.4 ± 8.7	86.0 ± 4.7	95.4 ± 7.1
Cycloheximide	93.0 ± 7.7	98.0 ± 11.3	0.0
Bafilomycin (100 nM)	88.7 ± 1.3	91.2 ± 7.2	60.3 ± 4.8
Flt3 inhibitor (1 μM)	76.1 ± 1.0	69.8 ± 1.3	88.7 ± 1.0
LY83583 (1 μM)	76.0 ± 2.6	81.1 ± 2.6	91.2 ± 1.3
Apomorphine (5 μM)	86.9 ± 1.0	78.6 ± 7.2	90.7 ± 4.2
CoCl_2_ (100 μM)	77.8 ± 1.1	72.9 ± 4.0	76.4 ± 6.8
BI-6C9 (10 μM)	84.7 ± 2.5	78.7 ± 8.1	90.9 ± 1.0

*HT22 cells were treated for 24 h with glutamate (5 mM), erastin (0.5 μM), or RSL3 (250 nM) alone (no treatment) or in the presence of the indicated concentrations of clorgyline, which inhibits mitochondrial ROS production (Tan et al., 1998a), the protein translation inhibitor cycloheximide, vacuolar H^+^-ATPase and autophagy inhibitor bafilomycin A, FMS-like tyrosine kinase 3 (Flt3) inhibitor III (Kang et al., 2014), the soluble guanylate cyclase inhibitor LY83583, inhibitors of calcium influx including the dopamine receptor D4 agonist apomorphine and cobalt chloride (CoCl_2_) and the Bid inhibitor BI-6C9. Cell survival was determined by the MTT assay and confirmed by visual inspection. Note that all compounds protect against oxytosis and ferroptosis induced by erastin. RSL3 also closely resembles these two compounds with the notable exception of cycloheximide*.

Induction of ferroptosis by erastin also induces ROS when measured by DCF fluorescence. Since antimycin A, a mitochondrial complex III inhibitor, partially suppressed erastin-induced cell death, it was concluded that mitochondria-triggered ROS production is essential for erastin-induced ferroptosis (Yagoda et al., 2007). Interestingly, cells with increased sensitivity to ferroptosis show an increased level of basal ROS production (Yang et al., 2014) associated with an increased iron content (Yang and Stockwell, 2008). Upon induction of GSH depletion by either erastin or BSO, both hydrophilic ROS detected by DCF and lipid peroxides detected by BODIPY 581/591 C11 accumulate in ferroptosis-sensitive cells (Yang et al., 2014). During cell death in response to inducible GPX4 inactivation in MEF, lipid peroxidation clearly preceded the accumulation of hydrophilic ROS (Seiler et al., 2008). Surprisingly, erastin sensitivity was preserved in KRAS mutant 143B osteosarcoma cells lacking mitochondrial DNA (mtDNA)-encoded transcripts, which led to the hypothesis that ferroptosis might occur independently of mitochondrial ROS production (Dixon et al., 2012). However, mitochondrial DNA has been shown to be dispensable for menadione-induced mitochondrial ROS production and the mitochondrial apoptosis machinery (Jacobson et al., 1993; Marchetti et al., 1996). In addition, FCCP, a compound that inhibits oxytosis by blocking mitochondrial ROS production (Tan et al., 1998a), also inhibits ferroptosis (Maher et al., 2017). Similarly, clorgyline also protects from both erastin and RSL3 toxicity in HT22 cells (Table 1). Furthermore, other compounds that target mitochondrial proteins have been shown to inhibit ferroptosis including the glutaminase inhibitor compound 968 (Gao et al., 2015).

Upon electron microscopic examination, cells treated with ferroptosis inducers show prominent alterations in mitochondrial morphology (Yagoda et al., 2007; Dixon et al., 2012; Friedmann Angeli et al., 2014; Doll et al., 2017; Guo et al., 2017; Figures 1A–C). Moreover, a robust increase in MitoSOX fluorescence, an indicator of mitochondrial H_2_O_2_ production, was observed in cells treated with erastin (Neitemeier et al., 2017). Similar to oxytosis, ROS from sources other than mitochondria may also contribute to ferroptosis. Indeed, using the highly specific fluorescent probe LiperFluo that directly interacts with (phospho)lipid hydroperoxides, robust (phospho)lipid peroxidation could be localized to the endoplasmic reticulum during ferroptosis induced by RSL3 (Kagan et al., 2017). It is well-known that the endoplasmic reticulum can be a significant source of ROS, accounting for ~25% of ROS production emanating from oxidative folding of proteins (Tu and Weissman, 2004). Finally, NOX1 activity has been reported to modulate lipid-ROS and the sensitivity to erastin in tumor cells (Xie et al., 2017).

Thus, the production of ROS, and in particular lipid peroxides, is an essential step in the cell death cascade for both ferroptosis and oxytosis. The contribution of different sources of ROS might vary between different cell types. Mitochondrial sources of non-lipid-ROS seem to be secondary to the generation of lipid hydroperoxides, the latter most probably generated by LOX activity. Whether LOX-induced changes of the mitochondrial outer membrane (van Leyen et al., 1998) directly induce mitochondrial non-lipid-ROS generation is currently unknown. The importance of lipid hydroperoxides as initiators of both ferroptotic and oxytotic cell death was underscored by the observation that the ferroptosis inhibitors ferrostatin-1, liproxstatin-1 as well as 1,8-tetrahydronaphthyridinol derivatives protect against ferroptosis induced by genetic GPX4 deletion in MEFs and glutamate-induced oxytosis in HT22 cells, correlating with their ability to prevent lipid peroxidation by trapping chain-carrying peroxyl radicals (Zilka et al., 2017).

## cGMP in oxytosis and ferroptosis

Activated ALOX15, which generates various metabolites like 12- and 15-HETE, might be involved in translating the increase in ROS into a calcium signal because soluble guanylate cyclase (sGC) is among the targets of these LOX metabolites (Brune and Ullrich, 1991). Inhibition of sGC by LY83583 blocks the execution of cell death by preventing the late calcium influx rather than GSH loss or ROS accumulation following glutamate treatment to initiate oxytosis (Li et al., 1997a). Correspondingly, cGMP levels peak after 6–8 h when GSH is depleted and ROS exponentially accumulate. In addition, the phosphodiesterase-resistant cGMP analog CPT-cGMP potentiates glutamate-induced oxytosis, even when added up to 8 h after treatment with glutamate (Li et al., 1997a). Finally, cell death similar to oxytosis can be induced by CPT-cGMP (Henke et al., 2013). Thus, elevated cGMP levels eventually contribute to the opening of calcium channels resulting in a detrimental influx of calcium and finally cell death. Of note, the sGC inhibitor LY83583 not only protects against glutamate-induced oxytosis but also against erastin and RSL3 (Table 1).

## Calcium in oxytosis and ferroptosis

During the exponential phase of ROS accumulation in oxytosis, there is a sharp increase in cellular calcium that immediately precedes cell death (Tan et al., 1998a). Of note, there is a mutual requirement for calcium and ROS for each to reach their maximal levels (Tan et al., 1998a). Even the first reports on the cell death pathway showed that oxytosis is efficiently inhibited by the calcium chelator EGTA in N18-RE-105 cells (Murphy et al., 1988). In addition, blockers of voltage-gated calcium channels (VGCC) such as CdCl_2_ completely prevented glutamate-induced oxytosis, while MnCl_2_ and LaCl_2_ reduced cell death by ~50% (Murphy et al., 1988). Moreover, although dihydropyridine calcium channel inhibitors had little effect, inhibitors of L-type and T-type VGCC (Bergson et al., 2011) protected against oxytosis (Murphy et al., 1988). Of note, the protective activity of the general calcium channel inhibitor CoCl_2_ against glutamate-induced oxytosis is retained until the very late stage of the cell death pathway indicating the functional relevance of the late calcium accumulation for cell death execution (Li et al., 1997a; Tan et al., 1998a). In addition, an inhibitor of store-operated calcium entry (SOCE), 2-aminoethoxydiphenyl borate, almost completely inhibited glutamate-induced oxytosis in HT22 cells with the same time profile as CoCl_2_ (Henke et al., 2013), indicating that SOCE might contribute to the final rise in cytosolic calcium during oxytosis. Indeed, siRNA-mediated knock-down of Orai1, but not of other components of SOCE such as STIM1, STIM2, and TRPM7 protected against glutamate-induced oxytosis and decreased peaks of intracellular calcium immediately before cell rupture (Henke et al., 2013). The fact that CPT-cGMP induces calcium peaks similar to those observed in glutamate-induced oxytosis before cell lysis and ORAI1 knock-down is similarly protective against CPT-cGMP-induced cell death suggests that cGMP regulates calcium influx in oxytosis. However, the exact mechanism of how cGMP modulates SOCE in oxytosis remains to be fully characterized. Of note, the influence of SOCE on oxytosis might either be cell type-specific or long-term SOCE deficiency induces more complex changes as fibroblasts derived from STIM1, STIM2, and ORAI1 knock-out mice were reported to have an increased sensitivity to glutamate-induced oxytosis (Henke et al., 2012).

In contrast, the role of calcium in ferroptosis has not been fully explored. While some reports suggest that calcium has no impact on ferroptosis (Wolpaw et al., 2011), several compounds that reduce calcium influx, including CoCl_2_ (Tan et al., 1998a) and apomorphine (Ishige et al., 2001a), protect cells against death induced by both of the functionally different ferroptosis inducers erastin and RSL3 (Table 1).

## Execution of cell death in ferroptosis and oxytosis

**Figure 2:**
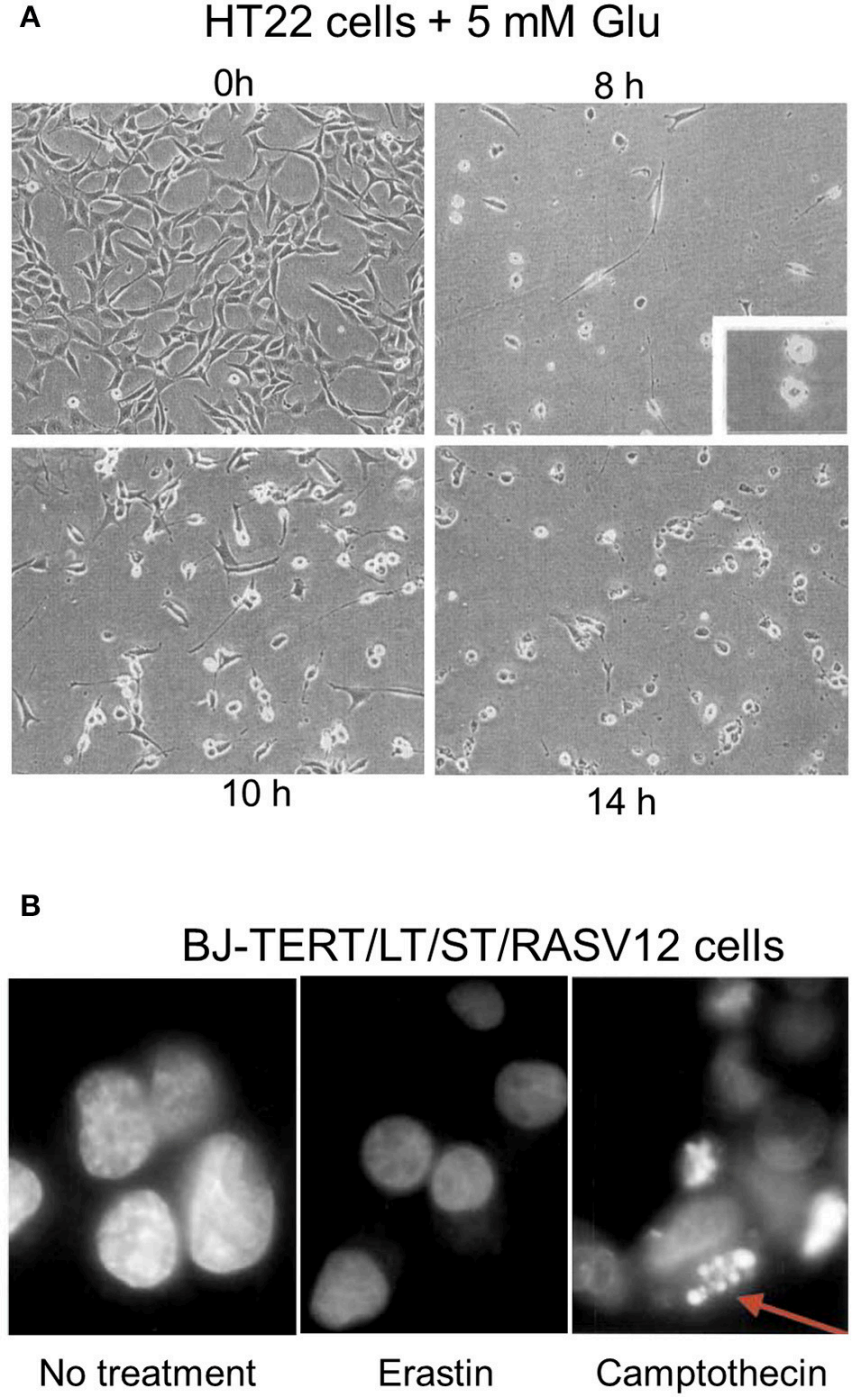
Light microscopic changes of cellular shape during oxytosis and the nucleus during ferroptosis. **(A)** HT22 cells exposed for indicated durations to 5 mM glutamate and observed by phase contrast microscopy. Cells were untreated (left upper) or treated with 5 mM glutamate for 8 h (right upper), 10 h (left lower), or 14 h (right lower) before examination under a phase-contrast microscope. Inset in the right upper shows blebs on surface of cells. **(B)** Lack of prominent nuclear changes in erastin-treated BJ-TERT/LT/ST/RASV12 cells while camptothecin-treated cells display fragmented nuclei (arrow). [Modified and reproduced with permission from **(A)** (Tan et al., 1998b) and **(B)** (Dolma et al., 2003)].

About 10–12 h after the induction of oxytosis, when both ROS and intracellular calcium levels have reached their maxima, the cells die (Tan et al., 2001b; Henke et al., 2013). The exact mechanisms underlying execution of cell death in oxytosis remain to be resolved. Ultrastructural examination revealed prominent morphological changes with swelling of the endoplasmic reticulum and the Golgi apparatus (Tan et al., 1998b; Figure 1D). Many cells appeared to contain large, cytoplasmic vacuoles associated with the rough endoplasmic reticulum and/or Golgi apparatus. In addition, the mitochondria, which retained both their outer and inner membranes, showed loss of their cristae. In contrast to these changes, the nucleus appeared largely unchanged without any signs of chromatin condensation or nuclear fragmentation, a hallmark of apoptosis (Tan et al., 1998b). Slightly different changes were observed in HT4 cells (Tirosh et al., 2000) where a prominent swelling of the mitochondria with cristaeolysis and outer membrane rupture was seen (Figure 1E). When observed by phase contrast light microscopy, the cells round up and first thin out their processes at ~8 h after glutamate exposure (Tan et al., 1998b; Lewerenz et al., 2003) followed by cell shrinkage and frequent membrane blebbing prior to disintegration of the cell (Figure 2A). Corresponding to the sparing of the nuclei based upon ultrastructural analysis, internucleosomal DNA fragmentation cannot be detected during oxytotic cell death in HT22 cells (Tan et al., 1998b). These observations are in line with the observation that caspase inhibitors that block apoptosis do not inhibit oxytosis (Tan et al., 1998b). In addition, the B-cell lymphoma protein 2 (Bcl-2) family member BAX has been excluded as part of the signaling cascade in oxytosis (Dargusch et al., 2001). BAX is transcriptionally upregulated by the tumor suppressor p53 (Selvakumaran et al., 1994) and upon death stimuli it dimerizes and inserts into the mitochondrial membrane to induce the mitochondrial pathway of apoptosis by mitochondrial outer membrane permeabilization (Cosentino and Garcia-Saez, 2017). Although spontaneous apoptosis was prominently decreased in primary neuronal cultures from BAX knock-out (BAX^−/−^) cells, no changes in the susceptibility to oxytosis were observed in immature BAX^−/−^ neurons compared to wild-type neurons (Dargusch et al., 2001). However, deficiency of another Bcl-2 family member, the anti-apoptotic Bcl-x_L_, is associated with increased susceptibility to oxytosis although these cells show a compensatory increase in cellular GSH, most probably via an activated pentose phosphate pathway (Pfeiffer et al., 2017). This increased sensitivity in response to the absence of Bcl-x_L_ was associated with fragmented mitochondria, reduced mitochondrial respiratory capacity and mitochondrial ATP content and augmented basal ROS production. The anti-apoptotic protein Bcl-2 has been found to be upregulated by stimulation of the cAMP pathway, which protects against oxytosis (Lewerenz et al., 2003; Sahin et al., 2006). Moreover, transient overexpression of Bcl-2 protects against oxytosis in HT22 cells while siRNA-mediated Bcl-2 knock-down sensitizes the cells by increasing ROS production (Sahin et al., 2006). However, the precise mechanisms whereby classical modulators of apoptosis impact sensitivity to oxytosis still need further investigation. Interestingly, a non-canonical role of Bcl-2 in ferroptosis has been described (see below) (Gascon et al., 2016) indicating that the effect of Bcl-2 family members on oxytosis might be independent of the anti-apoptotic functions of these proteins.

Another protein repeatedly reported to be involved in oxytosis is the pro-apoptotic AIF. AIF is a mitochondrial intermembrane protein that is released by mitochondrial outer membrane transition. In a caspase-independent manner, it causes large scale DNA fragmentation of ~50 kB, chromatin condensation and finally nuclear fragmentation, as well as mitochondrial swelling and dissipation of the ΔΨ_m_, and exposure of phosphatidylserine on the outer layer of the plasma membrane, all hallmarks of classical apoptosis (Susin et al., 1999). As release of AIF from the mitochondria can be prevented by Bcl-2 (Susin et al., 1999), this might explain the protective action of Bcl-2 in oxytosis. Moreover, calpain I has been shown to induce AIF release from the mitochondria and a prolonged increase of intracellular calcium, as present in oxytosis, can trigger calpain-mediated AIF translocation (Cao et al., 2007; Norberg et al., 2008). Calpain activation has been demonstrated during oxytosis in HT22 cells by some authors (Elphick et al., 2008). Some calpain inhibitors (ALLN and PD150606) have protective activity against oxytosis in HT22 cells (Elphick et al., 2008) while another (calpeptin) did not protect (Tan et al., 1998b). Thus, the role of calpains is still controversial.

Translocation of AIF has been observed during the execution of oxytosis by some investigators (Zhang and Bhavnani, 2006; Landshamer et al., 2008). The data published by Landshamer et al. support the idea that the truncated BH3-only domain protein Bid (tBid) translocates to the mitochondria, inducing AIF release and thereby cell death (Landshamer et al., 2008). Indeed, Bid can be processed to tBid directly by calpain (Cabon et al., 2012). This pathway from calcium increase to cell death via calpain activation, Bid processing and AIF release could explain the resistance of glutamate-induced oxytosis in HT22 cells to classical apoptosis inhibitors (Tan et al., 1998b; Landshamer et al., 2008).

However, it must be noted that there are some discrepancies that still need to be resolved until a role for AIF in oxytosis can be firmly established. Firstly, the Maher lab failed to detect large-scale DNA fragmentation, the indicator of AIF activity in the nucleus (Susin et al., 1999), during oxytosis (Tan et al., 1998b). Secondly, AIF was originally purified as a mitochondrial factor leading to chromatin condensation as well as nuclear fragmentation, hallmarks of classical apoptosis, in preparations of purified nuclei (Susin et al., 1996, 1999). In contrast, although some nuclear shrinkage might occur late in oxytosis, probably after the cells have disintegrated, the nucleus remains largely intact (Figures 1D,E) while prominent changes of the mitochondria and other organelles can be readily visualized by electron microscopy (Tan et al., 1998b; Figures 1D,E). Thus, in the absence of the two most important nuclear downstream effects of AIF, its involvement in oxytosis execution needs to be further characterized.

As reported for oxytosis, the structural changes during ferroptosis induced by erastin did not include changes in overt nuclear morphology characteristic of apoptosis (Dolma et al., 2003; Yagoda et al., 2007; Friedmann Angeli et al., 2014; Doll et al., 2017) although at late stages in the cell death process slight nuclear shrinkage was observed (Dolma et al., 2003; Seiler et al., 2008; Figure 2B). However, depending on the cell type, insult and the time of examination, prominent ultrastructural changes of the mitochondria were reported (Yagoda et al., 2007; Dixon et al., 2012; Friedmann Angeli et al., 2014; Doll et al., 2017) (Guo et al., 2017; Figures 1A–C). In line with the consistent lack of apoptotic changes of the nucleus, no cleavage-mediated activation of caspase 3 was observed during ferroptosis induced by erastin (Yagoda et al., 2007) or GPX4 depletion (Seiler et al., 2008), nor do caspase inhibitors protect (Seiler et al., 2008).

Interestingly, the cell death that occurs during direct astrocyte-to-neuron reprogramming is associated with lipid peroxidation and blocked by the ferroptosis inhibitors liproxstatin 1 and vitamin E, which thus might represent ferroptosis (Gascon et al., 2016). Transduction with Bcl-2 protects against this type of cell death indicating that Bcl-2 may also have a protective role in ferroptosis (Gascon et al., 2016). However, mutant Bcl-2 that cannot interact and sequester with the pro-apoptotic Bcl-2 family members BAX and BAK proved to be more efficient at preventing cell death than wild-type Bcl-2 and mutants that show increased BAX/BAK interaction. Thus, it was concluded that Bcl-2 in this context is protective in a non-canonical manner (Gascon et al., 2016). This finding is corroborated by the report that fibroblasts derived from BAX/BAK knock-out mice are equally susceptible to erastin-induced ferroptosis (Dixon et al., 2012). However, over-expression of Bcl-2 did not protect fibroblasts against induction of ferroptosis in response to GPX4 inactivation (Seiler et al., 2008). Thus, the role of Bcl-2 in ferroptosis might be cell type-specific, depending on the insult that induces cell death and/or on the type of Bcl-2 used. In addition, similar to the described role in oxytosis, nuclear translocation of AIF has been reported during ferroptosis induced by GPX4 inactivation with siRNA-mediated knock-down being protective (Seiler et al., 2008). In addition, Bid knock-out HT22 cells are similarly resistant against glutamate and erastin-induced cell death (Neitemeier et al., 2017) and the Bid inhibitor BI-6C9 protects from both oxytosis and ferroptosis induced by either erastin or RSL3 (Table 1).

For p53, a tumor suppressor that plays a critical role in the checkpoint control of cells in response to a wide spectrum of DNA damaging or stress signals and apoptosis (Zhan et al., 1994; Rosemary Siafakas and Richardson, 2009), opposing roles have been reported, either promoting or inhibiting ferroptosis. Perhaps, the effects of p53 are context-dependent. In addition, while it has been proposed that dysfunctional p53 leads to upregulation of the cystine/glutamate antiporter system ${\text{x}}_{\text{c}}^{-}$ in tumor cells and thereby to resistance against ferroptosis induced by oxidative stress (Jiang et al., 2015), pro-apoptotic non-transcriptional mechanisms induce sensitization of tumor cells to ferroptotic death in response to p53 deficiency by re-distribution of the dipeptidyl peptidase 4 to the plasma membrane and subsequent membrane lipid peroxidation (Xie et al., 2017).

## Metabolic changes and activation of signaling pathways during oxytosis and ferroptosis

The metabolic state of a cell has an important impact on its sensitivity to oxytosis. This is exemplified by the observation that cell density in proliferating cells is a critical regulator of oxytosis (Lewerenz et al., 2013). In addition, induction of eIF2α phosphorylation as an adaptive mechanism to adjust the cellular metabolism in response to limitations of amino acids potently protects against oxytosis (Tan et al., 2001b; Lewerenz and Maher, 2009; Lewerenz et al., 2012). Correspondingly, arginase, an enzyme that catalyzes the degradation of arginine in the culture medium, has been identified as anti-oxytotic (Esch et al., 1998). Arginase treatment induces ATF4 in primary embryonic neurons and protects against oxytosis brought about by the system ${\text{x}}_{\text{c}}^{-}$ inhibitor homocysteate (Lange et al., 2008) although the same authors showed that induction of ATF4 during activation of oxytosis acts as a pro-death regulator (Lange et al., 2008).

Interestingly, increasing serum in the culture medium strongly enhances the sensitivity of HT22 cells to oxytosis induced by glutamate (Maher and Davis, 1996). Serum contains many growth factors and signaling molecules that may activate signaling pathways in cells thereby modifying the sensitivity to oxytosis (e.g., signaling via PI3K protects by increasing eIF2α phosphorylation and ATF4 upregulation; Lewerenz et al., 2014). PI3K-mediated upregulation of SOD2 in response to resveratrol was also protective in oxytosis (Fukui et al., 2010). The pro-oxytotic factors in serum have not been investigated in depth.

Ferroptosis can be induced in MEF by amino acid deprivation in medium supplemented with serum (Gao et al., 2015). In this paradigm, cell death is induced by the absence of cystine in the medium which results in GSH depletion. The pro-death factors contained in serum in this paradigm were identified as transferrin, the carrier protein of iron, consistent with the view that iron is important in ferroptosis and, surprisingly, the amino acid glutamine. It was demonstrated that a product of glutamine metabolism, α-ketoglutarate, mediates the pro-death effect of glutamine. However, how α-ketoglutarate acts as a pro-ferroptotic factor remains to be resolved. Mitochondrial metabolism of α-ketoglutarate is associated with ROS production through α-ketoglutarate dehydrogenase and pyruvate dehydrogenase (Adam-Vizi and Starkov, 2010), indicating that increased levels of α-ketoglutarate might directly increase ROS. Another change in the metabolism of cells after the initiation of ferroptosis by erastin-mediated system ${\text{x}}_{\text{c}}^{-}$ inhibition is the accumulation of the GSH analog ophthalmic acid (Skouta et al., 2014). The production of ophthalmic acid is catalyzed by the same enzymes that are involved in GSH synthesis with the exception that α-aminobutyrate replaces cysteine. Ophthalmic acid, although redox inactive, may further decrease GSH synthesis by feed-back inhibition on GSH synthesizing enzymes (Kobayashi et al., 2017).

Another pathway that has been repeatedly reported to modify oxytotic cell death is the Ras-ERK pathway. However, the role of this pathway is still unclear. In 2000, two studies (Satoh et al., 2000; Stanciu et al., 2000) reported that ERK phosphorylation was increased in HT22 cells in response to glutamate treatment and that the MEK inhibitor U0126 could protect the cells from oxytosis. In contrast, a study published in 2001 (Maher, 2001) did not find an increase in glutamate-stimulated ERK phosphorylation in HT22 cells over the same time frame analyzed in the earlier studies and, more importantly, showed that two distinct MEK inhibitors, PD98059 and PD18452, did not prevent cell death. Interestingly, this latter observation was consistent with one of the earlier studies (Satoh et al., 2000), which also noted no effect of PD98059 on glutamate-induced cell death. In contrast, activation of ERK protected against oxytotic cell death (Maher, 2001). Both findings were recently replicated (Shibata et al., 2017).

Although the first study on erastin (Yagoda et al., 2007) found that ferroptosis could be completely blocked by MEK inhibition, a more recent study (Gao et al., 2015) has shown that this is likely due to the use of the MEK inhibitor U0126 in the earlier study. Unlike other MEK inhibitors, U0126 has anti-oxidant activity (Gao et al., 2015) and can protect cells against oxidative stress independently of its activity as an MEK inhibitor (Ong et al., 2015). Furthermore, UO126 also inhibits p70^S6K^, a kinase involved in protein translation (Fukazawa and Uehara, 2000). Modulation of translation has been repeatedly shown to protect against oxytosis and ferroptosis (Tan et al., 1998b; Yang and Stockwell, 2008; Dixon et al., 2012, Table 1 and see below). Thus, these results strongly suggest that any study that relies solely on U0126 is not reliable and that both oxytosis and ferroptosis do not require ERK activation. However, late ERK phosphorylation during oxytosis/ferrroptosis due to inactivation of phosphatases may occur in response to oxidative stress (Sen et al., 2012).

## Macromolecular synthesis during oxytosis and ferroptosis

Since inhibitors of both RNA and protein synthesis inhibit oxytosis in HT22 cells (Tan et al., 1998b), gene transcription and protein expression seem to be required for the execution of the cell death pathway. One of the genes induced during the course of oxytosis is GADD45α (Choi et al., 2011), which encodes a nuclear protein induced by p53. During oxytosis, c-Jun N-terminal kinase (JNK) induces GADD45α via p53 (Choi et al., 2011). Inhibition of JNK and knock-down of GADD45α block the execution of oxytosis (Choi et al., 2011). Of note, GADD45α functions as a heterochromatin relaxer (Chen et al., 2016) indicating that its expression might regulate additional genes important for the execution of oxytosis. However, others have argued that inhibition of protein synthesis simply reduces the consumption of cysteine, which would then be available for GSH biosynthesis (Ratan et al., 1994). In line with this assumption, phosphorylation of eIF2α, which is part of the multimeric eIF2 complex that is involved in the initiation of cap-dependent protein translation and the phosphorylation of which decreases cap-dependent translation (Wek et al., 2006), protects against oxytosis (Tan et al., 2001b; Lewerenz and Maher, 2009). However, under conditions that induced eIF2α-mediated cytoprotection, total protein synthesis was not perturbed in HT22 cells (Tan et al., 2001b). Moreover, the protection by eIF2α phosphorylation is mediated by cap-independent translation of the nuclear factor ATF4 and subsequent upregulation of xCT, the specific subunit of system ${\text{x}}_{\text{c}}^{-}$ (Lewerenz and Maher, 2009). In addition, the argument that macromolecular synthesis inhibitors protect HT22 cells against oxytosis by shunting cysteine from protein synthesis to GSH synthesis was contradicted by the observation that these compounds also protect against oxytosis induced by treatment with BSO (Tan et al., 1998b).

Similar to oxytosis in HT22 cells, erastin-induced ferroptosis in three different cell lines, HT-1080, BJeLR, and Calu-1 cells as well as fibroblasts and HT22 cells was consistently blocked by the protein synthesis inhibitor cycloheximide (Yang and Stockwell, 2008; Dixon et al., 2012; Table 1). Cycloheximide, however, neither protects oncogenic human foreskin fibroblasts (Yang and Stockwell, 2008) nor HT22 cells (Table 1) from RSL3-mediated GPX4 inhibition. These findings are fully consistent with protein synthesis being required early in the cell death pathway, downstream of inhibition of system ${\text{x}}_{\text{c}}^{-}$ (Tan et al., 1998b) but upstream of GPX4 inhibition.

However, the downstream targets whose synthesis is inhibited by cycloheximide and thereby mediate its protective activity still need to be fully characterized. Prostaglandin-endoperoxide synthase 2 (PTGS2) expression, which encodes cyclooxygenase-2 (COX-2), is prominently upregulated during piperazine erastin-induced ferroptosis (Yang et al., 2014), Targeted disruption of GPX4 in mouse skin *in vivo* (Sengupta et al., 2013) as well as GPX4 knock-down or its inhibition by RSL3 in tumor cells *in vitro* also induce robust PTGS2 expression (Seiler et al., 2008; Yang et al., 2014). However, COX-2 inhibition did not prevent erastin- or RSL3-induced ferroptosis (Sengupta et al., 2013). Thus, while COX-2 is not a cycloheximide target that mediates cell death, it might represent a marker for ferroptosis. Other transcripts highly upregulated in response to ferroptosis initiated by erastin include cation transport regulator-like protein 1 (CHAC1) (Dixon et al., 2014), a pro-apoptotic ER stress protein downstream of the pancreatic eIF2α-ATF4 pathway known to be upregulated by oxidized phospholipids (Mungrue et al., 2009), and DNA damage inducible transcript 4 (DDIT4) (Dixon et al., 2014), previously identified as part of the *in vivo* gene expression signature of oxidative stress (Han et al., 2008).

## Autophagy in ferroptosis and oxytosis

As noted above, there is evidence that lysosomes contribute to ROS production in oxytosis (Kubota et al., 2010). However, while this study showed that multiple autophagy inhibitors could reduce both glutamate-induced ROS production and cell death, no activation of autophagy was seen. In contrast, others demonstrated a clear induction of autophagy by glutamate as indicated by a time-dependent increase in autophagosome-bound LC3-II and loss of the LC3 binding protein and autophagy substrate p62 (Kim et al., 2009; Kumari et al., 2012).

Lysosomal ROS production was also recently shown to be involved in ferroptosis (Torii et al., 2016) where, identical to oxytosis, multiple autophagy inhibitors, such as bafilomycin A, blocked erastin- or RSL3-induced cell death. Similar results were obtained in HT22 cells (Table 1). This is in contrast to the original study on erastin that claimed that autophagy inhibitors did not prevent ferroptosis (Dixon et al., 2012) but is entirely consistent with several other recent studies that do show a requirement for autophagy in ferroptosis (Gao et al., 2016; Hou et al., 2016). Moreover, all of these recent studies have focused on the role of autophagy and lysosomes in modulating cellular iron levels since activation of autophagy is known to result in the turnover of the important iron-binding protein ferritin (a process known as ferritinophagy) (Biasiotto et al., 2016). Holoferritin is a multimeric protein comprised of 24 subunits consisting of both ferritin light polypeptide 1 (FTL1) and ferritin heavy polypeptide 1 (FTH1) and up to 4,500 molecules of Fe(III) in a redox inactive, inorganic form (Harrison and Arosio, 1996). However, during oxidative stress, iron can be released from ferritin in a redox active form, in part by copper-dependent mechanisms (Aliaga et al., 2011), inducing lipid peroxidation (Thomas et al., 1985). Thus, sequestering iron into holoferritin may be protective as illustrated by the observation that FTH1 is upregulated by Nrf2 in response to oxidative stress (Tsuji et al., 2000).

If redox active iron is released by ferritinophagy during the execution of oxytosis or ferroptosis, this should be associated with a decrease in ferritin protein subunits. However, the reports on this question are conflicting (Gao et al., 2016; Hou et al., 2016). This may be a reflection of different experimental conditions, release of iron in the absence of ferritin degradation or simply due to the focus on different ferritin subunits [FTL1 (Gao et al., 2016) vs. FTH1 (Hou et al., 2016)]. Consistent with these assumptions, transferrin has been identified as one of the serum factors that sensitizes cells to ferroptosis (Gao et al., 2015). Thus, there is ample evidence for a role for autophagy in both ferroptosis and oxytosis, probably involving ferritinophagy, but the precise nature of that role remains to be determined and may be cell-type dependent.

However, since there is a potential for autophagy inducers to activate ferroptosis/oxytosis (see above), it seems that this idea needs to be carefully evaluated both *in vitro* and *in vivo* before this therapeutic strategy is pursued further.

## Transition metals in ferroptosis and oxytosis—challenging the concept of iron-dependent cell death

Transition metals like iron can catalyze the oxidation of biomolecules by Fenton chemistry (Halliwell, 1992), thereby contributing to the generation of ROS. The brain is characterized by a relatively high iron content and a dependence on mitochondrial respiration (Behl, 1999), which has long been taken as the argument that oxidative stress is highly relevant in CNS diseases (Beal, 1996). Thus, an iron-dependent form of cell death might be especially important in CNS diseases. As described above, the term ferroptosis was chosen due to the fact that (i) iron chelators block this type of cell death (Dixon et al., 2012), (ii) introduction of oncogenic mutations that sensitize cells to this type of cell death are associated with an increased intracellular iron content (Yang and Stockwell, 2008) and (iii) most importantly, transition metals other than iron reportedly failed to exacerbate this type of cell death (Dixon et al., 2012). Additionally, in a lentiviral screen, PHKG2, the catalytic subunit of the PHK (phosphorylase kinase) complex, was identified as a positive regulator of ferroptosis sensitivity by upregulating the intracellular iron pool (Yang et al., 2016) and, as discussed above, ferritinophagy might contribute to ferroptosis and oxytosis by releasing redox active iron.

Similarly to ferroptosis, oxytosis in HT22 cells can be inhibited by iron chelators (e.g., Liu and Schubert, 2009; Kang et al., 2014) and exacerbated by different sources of iron (Kang et al., 2014; Maher, 2017). As an alternative to blocking Fenton chemistry, it has been suggested that iron chelation can protect neurons against oxytosis by inducing hypoxia-incuble factor 1 signaling (Zaman et al., 1999; Soucek et al., 2003).

Thus, both oxytosis and ferroptosis show the same dependency on iron, further suggesting that both pathways are highly similar. However, the whole concept of ferroptosis was recently challenged by a study demonstrating that copper, the other important transition metal involved in redox metabolism in biological systems, influences both glutamate-induced oxytosis as well as erastin-mediated ferroptosis in HT22 cells to a similar extent as iron (Maher, 2017). Thus, at least under certain conditions, transition metals other than iron have the potential to exacerbate ferroptosis.

## Gene expression patterns associated with oxytosis and ferroptosis resistance

HT22 cells selected for resistance against oxytosis show a prominent increase in catalase expression as well as in the activity of GSH synthesizing enzymes but no expression changes in genes classically involved in apoptosis. These cells proved to be cross-resistant against GSH depletion by GCL inhibition, inhibition of GSH reductase and organic hydroperoxides (Sagara et al., 1998).

In other, independently generated glutamate-resistant HT22 cells, multiple mechanisms of oxytosis resistance were identified including ATF4-mediated upregulation of system x_c_- (Lewerenz et al., 2006, 2012), cooperative interaction of glutamate transporters with system x_c_- (Lewerenz et al., 2012), changes in mitochondrial metabolism and structure associated with ineffective energy metabolism but with an increased ability to maintain the mitochondrial membrane potential (Pfeiffer et al., 2013), increased expression of the mitochondrial protein GDAP1 (Noack et al., 2012), increased usage of glucose via the pentose phosphate pathway (Pfeiffer et al., 2013), reduction of SOCE (Henke et al., 2013), upregulation of signaling via G protein-coupled receptors (Sahin et al., 2006; Dittmer et al., 2008) and upregulation of aldehyde dehydrogenase 3A1 as well as TIGR, a giant peroxisomal superoxide dismutase motif-containing protein (Toutzaris et al., 2010) also known as SZT2 (Frankel et al., 2009).

For ferroptosis, it has been reported that the level of expression of ASCL4 in part predicts sensitivity to this type of cell death (Yuan et al., 2016). Erastin-resistant DU-145 human prostate cancer cells are also resistant to the system ${\text{x}}_{\text{c}}^{-}$ inhibitors sulfosalicylic acid and sorafenib (Dixon et al., 2014). The most upregulated genes in these cells were members of the aldo-keto reductase family 1 (AKR1C1-3), enzymes that participate in the detoxification of toxic lipid metabolites (such as 4-hydroxynonenal) generated downstream of the oxidation of various polyunsaturated fatty acid species (Burczynski et al., 2001). Thus, both the regulation of membrane phospholipid metabolism, e.g., by ACSL4, and also detoxification systems affect ferroptosis sensitivity.

**Figure 3:**
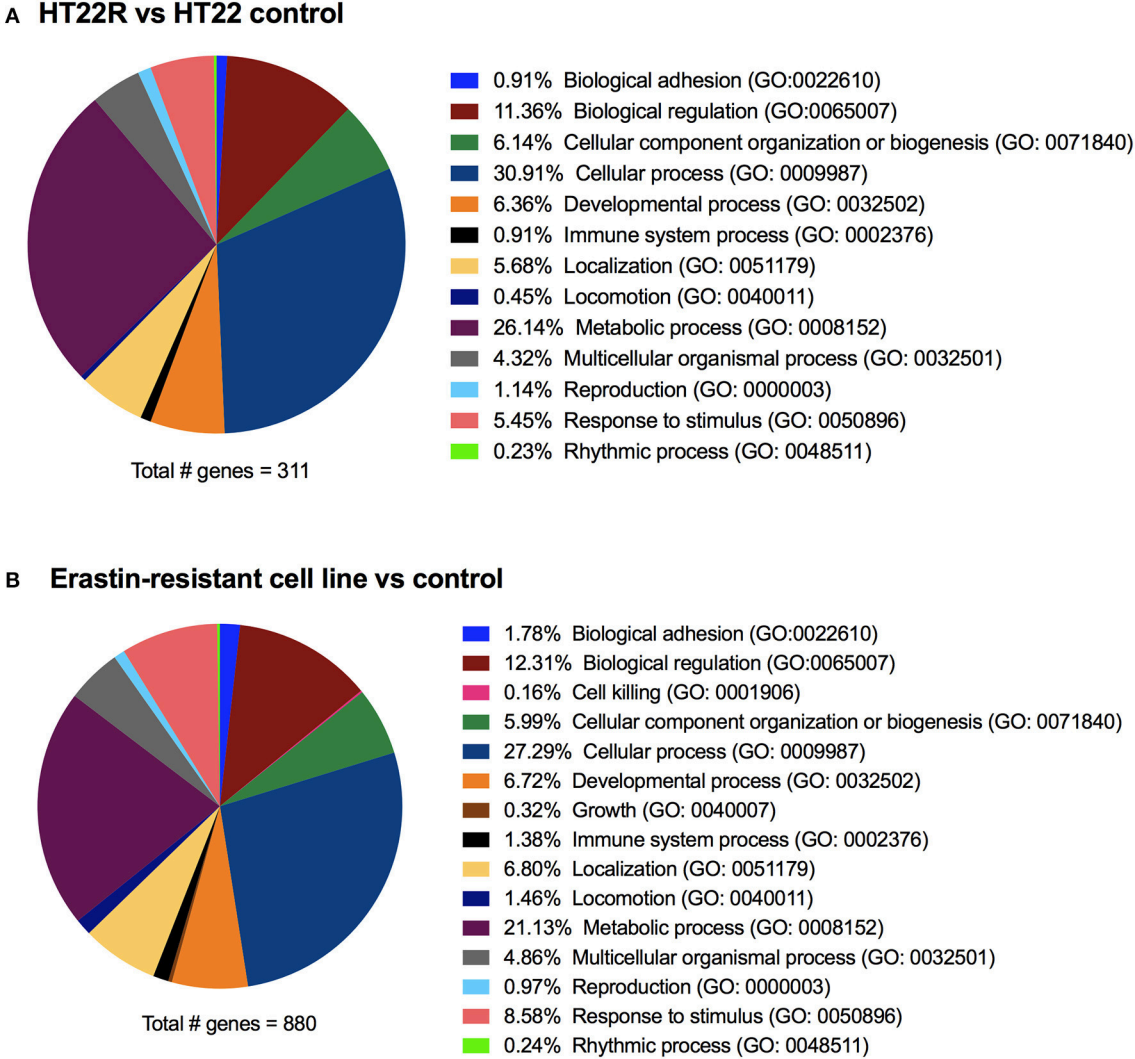
Transcriptome changes in acquired oxytosis and ferroptosis resistance. The transcriptomes of HT22 cells selected for resistance to oxytosis induced by glutamate (HT22R) **(A)** and DU-145 human prostate cancer cells selected for resistance against ferroptosis induced by erastin **(B)** (Dixon et al., 2014) were compared to their parental oxytosis/ferroptosis sensitive cell lines. Using the Panther classification system, deregulated genes (= fold-change ≥± 2) are grouped into biological processes. Both cell lines have highly similar deregulation profiles, even though the total number of deregulated genes differs greatly. The erastin-resistant cell line shows (minor) deregulation in only two additional processes compared to the HT22R. These processes (cell killing and cell growth) are likely to be attributed to the cancerous origin of this cell line. Gene Ontology (GO) identifications are shown between brackets.

To get a more comprehensive overview with regard to the mechanisms involved in ferroptosis and oxytosis resistance, we compared the transcriptome changes observed in glutamate-resistant vs. glutamate-sensitive mouse HT22 cells (A. Methner, unpublished results) with those generated from erastin-resistant and -sensitive human DU-145 cells (Dixon et al., 2014). Analysis using the Panther classification system (Mi et al., 2013) of both gene lists (output signal ≥100 and fold change ≥ ±2) showed, when grouping the genes into biological processes, highly similar changes in the overlapping groups in both HT22R and erastin-resistant cell lines (Dixon et al., 2014) when compared to their respective control cell lines (Figure 3).

## Summary

**Figure 4:**
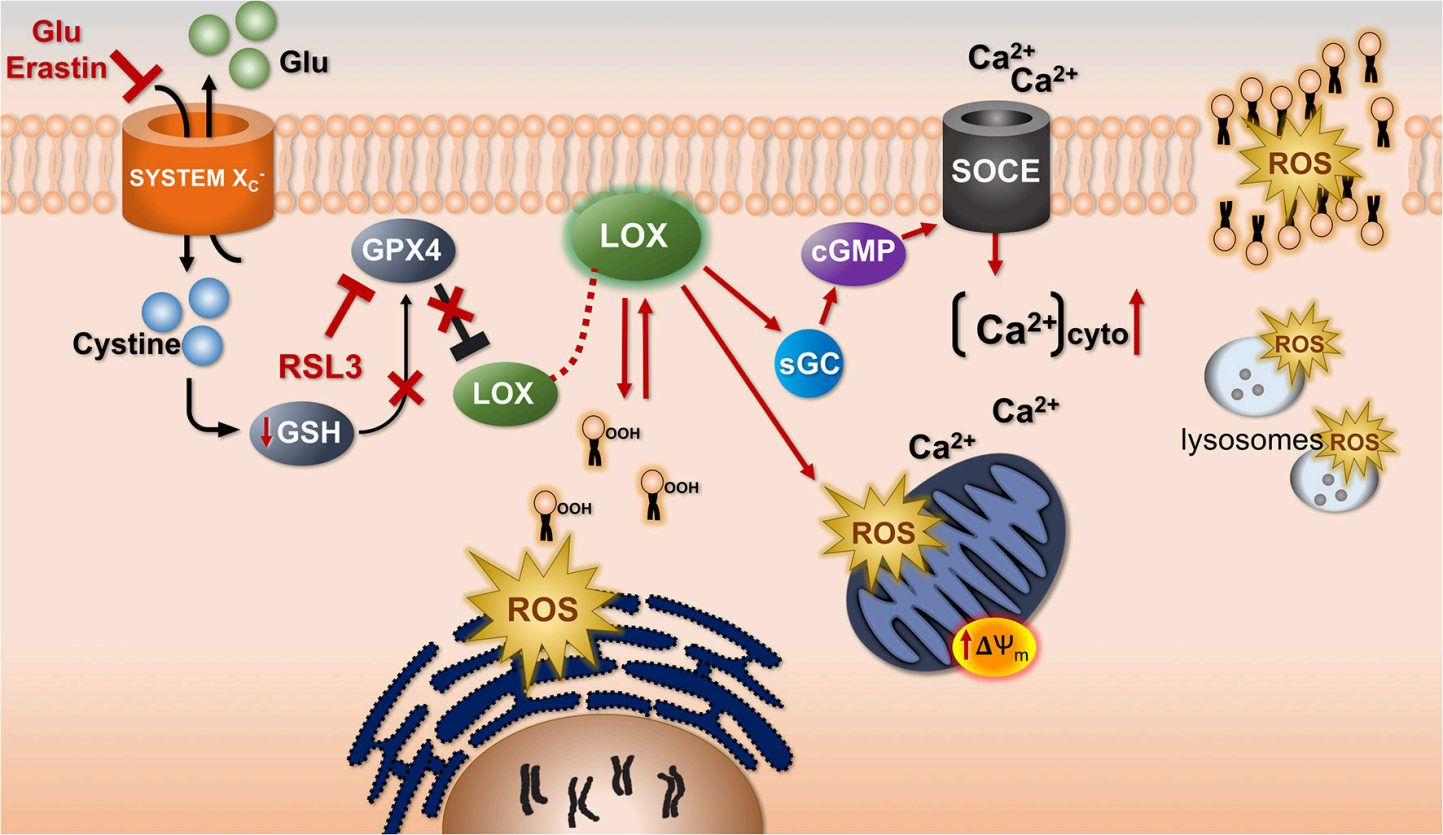
The common cell death pathway in oxytosis and ferroptosis. Uptake of cystine by system ${\text{x}}_{\text{c}}^{-}$ associated with counter-transport of glutamate (Glu) is inhibited by Glu and Erastin. This leads to depletion of glutathione (GSH) and subsequently inhibition of the GSH-dependent enzyme GSH peroxidase 4 (GPX4). GPX4 can also be directly inhibited by RSL3. GPX4 inhibition leads to activation of LOX. As a result, lipid hydroperoxides (lipid icons with OOH) accumulate probably in or very close to the endoplasmic reticulum as the initiating step of the production of reactive oxygen species (ROS). There is exponentially increasing mitochondrial ROS production associated with a hyperpolarization of the mitochondrial membrane potential (ΔΨm). Whether this is a direct effect of LOX is unknown. However, via activation of soluble guanylate cyclase (sGC) induced by LOX metabolites cGMP accumulates and calcium influx through store-operated calcium channels (SOCE) is activated. This increases cytoplasmic calcium (Ca^2+^)_cyto_. There is a mutual requirement for ROS and calcium to reach their maximal levels. Lysosomes also contribute to the overall ROS production.

Conclusively, the molecular pathways involved in the regulation of ferroptosis and oxytosis share many similarities (Figure 4). Even the molecules reported to induce ferroptosis and oxytosis are either the same in the case of BSO, or act via identical mechanisms, namely inhibition of cystine uptake by system ${\text{x}}_{\text{c}}^{-}$. In addition, the downstream players such as GPX4 and LOX and accumulation of mitochondria-derived ROS and nuclear translocation of AIF are identical. Moreover, transcriptomic changes in oxytosis and ferroptosis resistance correspond to identical pathways. Some characteristics have been studied in more detail under the name of either oxytosis or ferroptosis, e.g., the role of cGMP and calcium during oxytosis and the generation of lipid peroxides during ferroptosis.

In our opinion, the discrepancies that have been described in the scientific literature do not indicate that ferroptosis and oxytosis are different pathways of regulated cell death but rather result from methodological differences or cell type-specific variations on a single theme. Thus, oxytosis and ferroptosis should be regarded as two names for the same cell death pathway. As it has recently become clear than not only iron but also copper is a prominent metal regulator of oxytosis/ferroptosis (Maher, 2017), the term ferroptosis might be too narrowly conceived. However, that ROS play an important role in this type of cell death—as expressed by the term oxytosis—is generally accepted.

## Is oxytosis/ferroptosis relevant in diseases of the central nervous system?

### Non-apoptotic regulated cell death in human brain and animal models of neurological disease

Terminal transferase dUTP nick-end labeling (TUNEL) (McCarthy and Evan, 1998), a histochemical marker of DNA fragmentation and therefore cell death, has been reported in many diseases of the CNS including AD (reviewed by Behl, 2000), HD (Portera-Cailliau et al., 1995), ALS (Ekegren et al., 1999), and ischemic stroke (Endres et al., 1998). However, in AD TUNEL-positive cells were found to be only inconsistently associated with other structural features of apoptosis (reviewed by Behl, 2000). Correspondingly, increased neuronal nuclear localization of AIF was demonstrated in the brain regions most severely affected by AD pathology (Yu et al., 2010; Lee et al., 2012). *In vitro*, Aβ has been found to induce both necrosis (Behl et al., 1994) and apoptosis (Forloni et al., 1993) in neuronal cells. Interestingly, iron has been reported to be upregulated in the cortex of patients with AD upon high-resolution MRI *in vivo* (van Rooden et al., 2015) and *ex vivo* as well as in histochemical stainings (Bulk et al., 2018).

In cerebral ischemia, regulated cell death is thought to predominate in ischemic border zones, the so-called penumbra, in part by glutamate excitotoxicity (reviewed in Dirnagl et al., 1999). The detection of internucleosomal DNA fragmentation and the reduction in tissue damage by caspase inhibitors indicate that, at least in part, classical apoptosis is triggered by cerebral ischemia (Endres et al., 1998). However, AIF translocation has also been reported following cerebral ischemia (reviewed in Ferrer and Planas, 2003). Correspondingly, reduced AIF expression (Culmsee et al., 2005) has been shown to mitigate ischemic cell death in animal models of stroke. Moreover, excitotoxic neuronal cell death induced by glutamate *in vivo* is to some extent mediated by oxytosis *in vitro* (Schubert and Piasecki, 2001).

Transient global cerebral ischemia in rodents is a model of neuronal damage in response to hypoxia or cardiac arrest in humans. Global ischemia results in selective delayed neuronal death with region-specific differences in hypoxia sensitivity (reviewed in Schmidt-Kastner, 2015). When mild, it predominantly results in a delayed degeneration of the neurons of the CA1 subregion of the hippocampus. There has been a decades-long debate whether delayed neuronal degeneration in response to global ischemia represents necrosis, apoptosis or a type of delayed cell death that combines elements of both (reviewed in Martin et al., 1998; Schmidt-Kastner, 2015). However, the ultrastructural changes in neurons in response to global ischemia are very different from neuronal apoptosis (Martin et al., 1998). In addition, hypoxia-induced delayed neuronal cell death is associated with increased lipid peroxidation (Perez Velazquez et al., 2006) and activation of LOXs (Yigitkanli et al., 2017). Biphasic mitochondrial ROS production was reported which can be inhibited by antioxidants and iron chelation in association with neuroprotection (Park et al., 2011). Calpain I activation as well AIF have are reported to play a role (Cao et al., 2007), and calcium chelation (Calderone et al., 2004) and inhibition of SOCE (Zhang et al., 2014) protect. Execution of delayed neuronal cell death is associated with upregulation of PTGS2 mRNA and COX-2 protein (Nakayama et al., 1998). Furthermore, accumulation of blood-derived iron has been observed in post-hypoxic brain, which contributes to delayed oxidative stress and neuronal cell death (Park et al., 2011). Finally, this type of cell death depends on macromolecular synthesis as inhibitors of protein translation as well as gene transcription protect (Goto et al., 1990; Shigeno et al., 1990; Deshpande et al., 1992). In summary, while suggestive, whether delayed hypoxic neuronal death represents oxytosis/ferroptosis requires further investigation.

PD is characterized by a progressive degeneration of midbrain dopaminergic neurons in the substantia nigra (SN) and iron accumulation and GSH loss have been repeatedly observed in autoptic PD SN (Dexter et al., 1989; Jellinger et al., 1990; Sian et al., 1994; Fitzmaurice et al., 2003). Moreover, iron levels, as detected by magnetic resonance imaging, are upregulated in presymptomatic stages of PD (Pyatigorskaya et al., 2015) and pilot data indicate that iron chelation might be therapeutically beneficial in PD patients (Devos et al., 2014). The fact that dopamine metabolism is linked to the production of ROS (Anderson et al., 2011) and surrogate markers of oxidative stress are present in PD (Buhmann et al., 2004) indicates the potential pathophysiological relevance of oxidative stress in PD. Moreover, the differential susceptibility of SN dopaminergic neurons depending on their calcium buffering capacity as mediated by calbindin expression (Yamada et al., 1990) has led to the view that calcium might contribute to dopaminergic neuronal death in PD. Thus, iron, GSH, oxidative stress and calcium might all contribute to neurodegeneration in PD, an observation highly suggestive for an involvement of oxytosis/ferroptosis.

In transgenic mice expressing mutant SOD1 (the classical animal model for ALS), caspase 1 and 3 activation have been reported, and consequently caspase inhibition was shown to reduce motor neuron death (Li et al., 2000; Chi et al., 2007). In contrast, another study reported that motor neuron degeneration in SOD1 mutant mice resembles slow necrosis without evidence of caspase activation (Martin et al., 2007). Additionally, astrocytes derived from familial ALS patients are able to induce motor neuron cell death via Ripk1 dependent necroptosis *in vitro* (Re et al., 2014) and necroptosis plays a role in axonal degeneration in ALS *in vivo* (Ito et al., 2016). However, AIF nuclear translocation is seen in motor neurons from patients with sporadic ALS (Shibata et al., 2009), as well as in SOD1 mutant transgenic mice (Oh et al., 2006; Chi et al., 2007). Of note, spinal cord iron accumulation has been reported in mouse models of ALS, mutant SOD1^G37R^ (Jeong et al., 2009) and SOD1^G93A^ (Lee et al., 2015) mice. In human ALS, high-resolution MRI revealed iron accumulation in the motor cortex *in vivo* and well as in autoptic brain, in the latter confirmed by iron histochemistry (Kwan et al., 2012).

Ultrastructural analysis of both brain tissue from HD patients, a neurodegenerative disease caused by a CAG repeat expansion in the huntingtin gene (HTT), and mice expressing the first exon of HTT with increased CAG repeats showed shrunken, degenerating neurons, which did not display the nuclear fragmentation typical of apoptosis (Turmaine et al., 2000). Another study described upregulation of PARP1 in HD brains, whereas activated caspase 3 was only rarely observed (Vis et al., 2005). Fibroblasts derived from HD patients are more susceptible to oxytosis (Archer and Mancall, 1983), and ferroptosis inhibitors rescue mutant HTT-induced cell death in cellular HD models (Skouta et al., 2014). Last but not least, also in HD there is abundant iron accumulation, especially in the basal ganglia, correlating with disease severity (Penney et al., 1997; Dominguez et al., 2016).

Despite this contradictory evidence, huge numbers of studies have suggested that apoptosis is a major hallmark of neurodegeneration. The vast majority of these studies have relied solely on the detection of TUNEL-positive cells. However, since TUNEL staining does not allow one to discriminate between the internucleosomal DNA cleavage that is typical of apoptosis and large-scale DNA fragmentation as induced by AIF or EndoG and seen in other regulated cell death paradigms, unequivocal assignment of apoptotic or non-apoptotic regulated cell death in diseased brain is challenging (Charriaut-Marlangue and Ben-Ari, 1995). The issue is further complicated by the lack of specific markers for the recently described non-apoptotic forms of regulated cell death with the exception of necroptosis (staining for phospho-MLKL in human cells and tissues; Sun et al., 2012).

In light of the only very recent recognition of non-apoptotic forms of regulated cell death in brain diseases and the limitation of TUNEL staining for apoptotic cell death, it is conceivable that some, if not to say the majority, of these processes proceed through non-apoptotic cell death paradigms. Interestingly, there is ample evidence that oxidative stress plays an important role in ALS (Barber and Shaw, 2010), AD (Bonda et al., 2010), ischemic stroke (Shivakumar et al., 1995), and HD (Stack et al., 2008). In addition, iron accumulation occurs in ALS, AD, PD, and HD in brain regions affected most in the individual diseases (Kwan et al., 2012; Pyatigorskaya et al., 2015; van Rooden et al., 2015; Dominguez et al., 2016; Bulk et al., 2018) as well as in the posthypoxic brain (Park et al., 2011). Thus, novel types of regulated non-apoptotic cell death that encompass oxidative stress as a critical step and are facilitated by transition metals (i.e., oxytosis/ferroptosis) are strong candidates and are possibly highly relevant for many neurodegenerative diseases.

## Evidence for *in vivo* relevance of oxytosis/ferroptosis

### Genetic models for induction of oxytosis/ferroptosis

The majority of research that has addressed the role of oxytosis/ferroptosis *in vivo* has relied on inducible genetic GPX4 deletion either in whole animals or specific tissues (Angeli et al., 2017). Global, inducible GPX4 deletion resulted in acute renal failure and death within two weeks (Friedmann Angeli et al., 2014). This was associated with increased levels of lipid peroxidation in both mitochondrial and extra-mitochondrial compartments and could be delayed by treatment with the oxytosis/ferroptosis inhibitor liproxstatin-1. In a similar model of global inducible GPX4 deletion, rapid death was associated with neuronal loss restricted to the CA1 region of the hippocampus (Yoo et al., 2012). In contrast, in a neuron-specific non-inducible knock-out mouse, neuronal loss was most prominent in the CA3 region of the hippocampus and involved mainly GABAergic interneurons (Seiler et al., 2008). Neuronal loss was associated with intense astrogliosis (Seiler et al., 2008; Yoo et al., 2012) indicating that oxytotic/ferroptotic death is—in stark contrast to apoptosis—pro-inflammatory. Forebrain neuron-specific conditional deletion of GPX4 did not lead to rapid death but rather a delayed loss of hippocampal neurons in the absence of detectable caspase activation, which was associated with progressive deficits in spatial learning and memory (Hambright et al., 2017). Neuronal death was exacerbated by vitamin E deficiency, while liproxstatin-1 blunted this effect. Surprisingly, inducible global neuron-specific GPX4 deletion in adult mice caused rapid death due to spinal motor neuron degeneration, while neurons in the cerebral cortex remained unaffected at the time of death (Chen et al., 2015). Interestingly, conditional deletion of GPX4 in hypothalamic neuronal subpopulations [proopiomelanocortin (POMC) and agouti-related protein (AgRP) positive neurons] as well as in dopaminergic neurons did not result in overt neuronal loss (Schriever et al., 2017). In summary, the induction of oxytosis/ferroptosis by genetic GPX4 deletion relies on the timing of the deletion and whether it is restricted to specific cell types, e.g., neurons. In addition, dietary content of vitamin E might also modify the phenotype of GPX4 deletion.

In contrast to the genetic deletion of GPX4, deletion of xCT is not associated with an overt phenotype (Sato et al., 2005) and although xCT is expressed in the brain (Shih et al., 2006), no increase in oxidative stress or neuronal loss has been observed in these mice (De Bundel et al., 2011). This is in stark contrast to xCT-deficient cells in cell culture, which rapidly die from oxytosis/ferroptosis when antioxidants are omitted from the culture medium (Sato et al., 2005). This paradox is easily explained by the fact that cells in culture strongly rely on cystine in the culture medium since cysteine is readily oxidized to cystine in regular culture conditions (Lewerenz et al., 2013). *In vivo*, the oxygen concentrations in tissues are much lower and thus uptake of reduced cysteine can compensate for the lack of cystine uptake by system ${\text{x}}_{\text{c}}^{-}$ (Sato et al., 2005). Correspondingly, no overt phenotype of pharmacological system ${\text{x}}_{\text{c}}^{-}$ inhibition was observed after subcutaneous or intravenous delivery of erastin or its more stable analog piperazine erastin in athymic nude mice, although tumor growth was retarded indicating that *in vivo* tumor cells are sensitive to oxytotic/ferroptotic cell death induced by system ${\text{x}}_{\text{c}}^{-}$ inhibition (Yang et al., 2014). However, it remains unknown whether and to what extent erastin and piperazine erastin cross the blood-brain barrier. Thus, with the exception of tumor cells, oxytosis/ferroptosis *in vivo* is more likely to be induced by GPX4 inhibition rather than system ${\text{x}}_{\text{c}}^{-}$ inhibition.

## Pharmacological evidence for oxytosis/ferroptosis in CNS diseases

Since ferrostatin-1 and liproxstatin-1 protect against erastin- and RSL3-induced oxytosis/ferroptosis, but not against staurosporine-induced apoptosis or necroptosis (Dixon et al., 2012; Friedmann Angeli et al., 2014), they have been employed to demonstrate that ferroptotic/oxytotic neuronal death occurs in *in vitro* and *in vivo* models of neurological diseases.

Ballistic transfection of rat striatal slices with mutant HTT exon 1 induced loss of medium spiny neurons expressing mutant HTT that could be inhibited by ferrostatin-1 (Skouta et al., 2014). In addition, ischemic neuronal death induced by transient middle cerebral artery occlusion, a stroke model in mice, was substantially reduced by liproxstatin-1 (Tuo et al., 2017). Markers of ferroptosis, including increased lipid hydroperoxides, decreased GSH, decreased GPX4 activity and upregulation of PTGS2 are present in association with neuronal cell death in organotypic hippocampal slice cultures treated with hemoglobin, an *in vitro* model of intracerebral hemorrhage (ICH) (Li et al., 2017). In addition, neuronal death could be inhibited by ferrostatin-1. Similar observations were made in animal models of ICH suggesting that ferroptosis at least in part explains the delayed neuronal death that occurs after this insult.

With regard to PD, it was reported recently that human differentiated midbrain neurons are uniquely sensitive to oxytosis/ferroptosis brought about by erastin treatment in comparison to insults that induce apoptotic or autophagic cell death (Do Van et al., 2016). Moreover, both ferrostatin-1 and the iron chelator deferiprone reduced dopaminergic degeneration in animal models of PD (Devos et al., 2014; Do Van et al., 2016) and improved motor function in PD patients (Devos et al., 2014).

## Future perspectives

In summary, multi-layered evidence strongly suggests that oxytosis/ferroptosis plays an important role in CNS diseases. We conclude that oxytosis and ferroptosis are one single pathway. Combining accumulated research performed under both terms will therefore accelerate our understanding of this type of cell death. Most importantly, more specific tools are needed to unequivocally demonstrate where and when neurons undergo oxytosis/ferroptosis in different CNS pathologies. This will provide the necessary framework for clinical trials with oxytosis/ferroptosis inhibitors that are under development for treatment of neurodegenerative diseases.

## Author contributions

JL: provided the concepts for the review and wrote much of it; PM: wrote a portion of the review and revised the manuscript critically for important intellectual content; GA: wrote parts of the manuscript, performed the gene expression analysis, revised the manuscript critically for important intellectual content; MC and AM: contibuted substatiantially to the conception of the manuscript and revised it critically for important intellectual content.

### Conflict of interest statement

The authors declare that the research was conducted in the absence of any commercial or financial relationships that could be construed as a potential conflict of interest.
